# Solution blow spinning of polymer/nanocomposite micro-/nanofibers with tunable diameters and morphologies using a gas dynamic virtual nozzle

**DOI:** 10.1038/s41598-019-50477-6

**Published:** 2019-10-04

**Authors:** Ramakrishna Vasireddi, Joscha Kruse, Mohammad Vakili, Satishkumar Kulkarni, Thomas F. Keller, Diana C. F. Monteiro, Martin Trebbin

**Affiliations:** 1 The Hamburg Center for Ultrafast Imaging (CUI), University of Hamburg, Luruper Chaussee 149, 22761 Hamburg, Germany; 2 Donostia International Physics Center (DIPC), Manuel Lardizabal Ibilbidea 4, 20018 San Sebastian, Spain; 3 Deutsches Elektronen-Synchrotron (DESY), 22607 Hamburg, Germany; 4 Department of Physics, University of Hamburg, 20355 Hamburg, Germany; 5 Department of Chemistry, The State University of New York, University at Buffalo, 760 Natural Sciences Complex, Buffalo, New York, 14260-3000 USA

**Keywords:** Synthesis and processing, Polymers, Design, synthesis and processing

## Abstract

Uniform endless fibers are ubiquitous and their applications range from functional textiles over biomedical engineering to high-performance filtering and drug delivery systems. Here, we report a new method for the direct, reproducible fabrication of uniform polymer and composite micro-/nanofibers using a microfluidic gas flow focusing nozzle (Gas Dynamic Virtual Nozzle (GDVN)) relinquishing the need for external fiber pulling mechanisms. Compared to other methods, this technique is inexpensive, user-friendly and permits precise fiber diameter control (~250 nm to ~15 µm), high production rate (m/s-range) and direct fiber deposition without clogging due to stable, gas-focused jetting. Control over shape (flat or round) and surface patterning are achieved by simply tuning the air pressure and polymer concentration. The main thinning process happens after the polymer exits the device and is, therefore, mostly independent of the nozzle’s internal geometry. Nevertheless, the lithography-based device design is versatile, allowing for precise flow-field control for operation stability as well as particle alignment control. As an example, we demonstrate the successful production of endless hematite nanocomposite fibers which highlights this technology’s exciting possibilities that can lead to the fabrication of multifunctional/stimuli-responsive fibers with thermal and electrical conductivity, magnetic properties and enhanced mechanical stability.

## Introduction

Whether in nature or modern industry, micro-/nanofibers are ubiquitous because of their unique properties and utility. On account of their high specific area, surface roughness and strong interfacial interactions, micro-/nanofibers are already applied in textile fabrics, as reinforced materials in tissue engineering, high performance filters and as optical sensors^1–6^. A variety of techniques can be used for the fabrication of continuous (or endless) microfibers such as melt spinning, wet spinning, coaxial spinning, electrospinning and blow spinning^2–6^. However, most fibers generated by these techniques suffer from shape- and size-nonuniformity. Furthermore, manufacturing problems such as clogging of the hardware, complex setups and low-throughput pose industrial obstacles. Therefore, the fabrication of continuous submicron/nanofibers with tunable morphologies and uniformity remains a great challenge^7–12^.

Electrospinning is the only well-developed technique for fabricating nanofibers, making it the current state of the art manufacturing process^5,13,14^, even though it has a number of major drawbacks. Electrospinning requires hazardous operating conditions such as high voltages and in most cases the use of toxic and volatile organic solvents. The manufacturing conditions are also highly susceptible to local environmental changes, such as temperature or humidity, which can make reproducibility an enormous challenge. Thus, all of these factors need to be carefully controlled and have called for the development of new techniques for nanofiber spinning. One such technique is solution blow spinning, which also allows the fabrication of fibers in the nm-range without the need of a high voltage gradient, which can be advantageous when working with cells or other bio-systems^15–17^. Additionally, this fabrication process is performed under atmospheric pressure, does not involve harsh chemical conditions and can be carried out at room temperature^18^. Nevertheless, blow spinning is still in the state of initial development^19^ and fabrication of customized nozzles is challenging. One possible way around this difficulty is to employ well-established soft lithographic techniques used in the fabrication of microfluidic devices to facilitate nozzle production. Microfluidic approaches allow for fast prototyping and easy mass production of the nozzles. Furthermore, the miniaturized nature of the device reduces the needed space for a spinning setup and allows easy nozzle parallelization for high throughput operation with excellent reproducibility^7^.

Several fibers have been successfully spun using microfluidic techniques, but to our knowledge, single fibers with controlled diameters in the nm-range have not yet been achieved via the here-presented microfluidic approach^7,20–22^. Developing a new method for spinning fibers using Gas Dynamic Virtual Nozzles (GDVN) microfluidic devices allows the combination of advantages from coaxial spinning, electrospinning and blow spinning, while minimizing the current drawbacks of the individual techniques. GDVNs have been developed to allow the formation of free-standing liquid jets by gas-flow-focusing liquid samples^23^. The main advantage of this GDVN-technique is that the liquid does not contact the nozzle exit surface which allows for smooth, reproducible and continuous operation of the nozzle for long periods of time by avoiding the deposition of material at the nozzle exit.

Very recently, Hofmann *et al*. demonstrated the first use of a microfluidic GDVN for fiber spinning^7^, obtaining continuous fibers in the micrometer range by gas flow-focusing in a nozzle and coupled with fiber pulling. The fiber pulling step spools the fiber onto a surface and the spooling speed determines the final fiber diameter. Here, we demonstrate the development of a different microfluidic GDVN nozzle capable of continuously producing fibers down to nm-diameters without the need for external fiber spooling. Its reliable operation was made possible through our optimized gas flow-focusing geometry with very stable jetting regimes over large ranges of jetting conditions. This geometry also enables the production of fibers with a diverse range of surface and morphology characteristics, while maintaining a small diameter, not easily achieved by previously described methods^16^. Furthermore, nanocomposite fibers were also produced and shown to be easily accessible with this nozzle geometry, with the microfluidic design relieving problems such as clogging from the nanoparticle flow.

GDVN-based micro-/nanofiber spinning has the potential to simplify current production processes and minimize the required space and equipment for manufacture. Furthermore, due to the applied rapid prototyping approach inherent to UV- and soft-lithographic techniques, nozzles with different characteristics can be easily designed and fabricated and even arrayed to meet fiber production demands^7^. The highly uniform fibers produced in this work are very versatile for different applications such as air filtering units^24^, protective clothing^25^, biomedical engineering^26^ and many more^27–34^.

## Results and Discussion

### A microfluidic GDVN for fiber spinning

A GDVN-based microfluidic spinning device was produced with a geometry based on the previously described soft-lithography GDVN designed by Trebbin *et al*.^35^. The spinning procedure was easily carried out under non-hazardous conditions at room temperature and atmospheric pressure. A polymer solution in acetone is fed through a liquid inlet which is then accelerated by 3D flow-focusing with compressed air at the nozzle (Fig. S2). The gas envelops and pulls the emergent polymer jet from the nozzle, which rapidly dries to form a continuous fiber. The mechanics of the drying process are discussed in a later section. Such a gas-focusing approach also alleviates the *need* for *external* pulling forces, i.e. from rotational or counter-charged collectors, and therefore allows for the direct solution blow spinning of fibers or micro-/nanofiber deposition onto (non-charged) surfaces.

**Figure 1:**
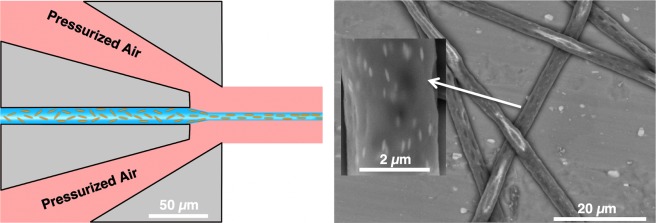
(*Left*) Schematic view of the experimental microfluidic gas-dynamic fiber spinning setup where the extensional flow along the fiber axis fosters parallel particle alignment. (*Right*) The corresponding element-sensitive backscattered electron (BSE)-SEM image of 2.5 wt% hematite-loaded microfibers shows the parallel alignment of well-dispersed anisotropic nanoparticles along the fiber axis.

The original liquid jet-purposed GDVN design^35^ was adapted and optimized to one for fiber spinning applications (Fig. S3B,C). These include changes of the distance from main channel to the nozzle outlet (now 35 µm vs the previous 95 µm) as well as a wider nozzle orifice (55 µm vs 30 µm). The shorter distance between the liquid channel and the nozzle orifice decreases the gas flow-focusing volume and the chances of clogging as the emergent fiber can quickly exit the device. The geometry of the nozzle designed for this work also differs significantly in the gas flow-focusing region compared to that described by Hofmann *et al*.^7^. While Hofmann *et al*. described a flow-focusing nozzle geometry in a perpendicular (90°) configuration (Fig. S3A), we chose an incident angle of the gas flow onto the liquid of only 15° (Fig. S3C).We believe that our shallower angle allows for a more forward-directed momentum transfer of the gas accelerating the liquid, resulting in increased flow alignment, smaller fibers and more stable flow focusing conditions^36,37^. This geometry allows us to produce thin endless fibers under a wide range of jetting conditions and without clogging. We were able to effectively and robustly spin THV polymer (a fluorinated terpolymer of tetrafluoroethylene, hexafluoropropylene and vinylidene fluoride monomers) micro-/nanofibers with highly tunable morphologies which extend beyond the circular and beaded morphologies described by Hofmann *et al*.^7^. The stable jetting also allowed for the fabrication of nanocomposite fibers impregnated with spindle-shaped hematite nanoparticles. Due to the flow-induced converging flow rapidly followed by fiber fixation, this anisotropic nanocomposite material was strongly aligned parallel to the fiber axis promising improved fiber mechanics^37,38^, as later shown in Fig. 1.

### Influences on fiber diameter

THV fibers of diameters ranging from ~250 nm to ~15 µm were produced. It is a commonly known fact that the fiber diameters can be influenced by the polymer content (final volume after solvent evaporation) as well as the drawing speeds and ratios in a jet-and-spool configuration^7^. Since our GDVN did not employ a fiber spooling process, the speed of the emergent fiber is solely determined by the liquid flow rate and pressure-gradient acceleration at the gas flow-focusing region. A wide range of jetting conditions could be employed: 10–25 wt% THV solutions in acetone, 0.5–2 bar of pressurized air (see Fig. S8 for gas flow rates) and 100–3000 µL/h polymer flow rates. The thinnest fibers fabricated were ~10x thinner than those previously reported^7^. Only a few techniques, such as e.g. solution blow spinning or electrospinning, which however requiring more complex experimental set-ups, have been previously shown to yield such thin, continuous single fibers.

**Figure 2:**
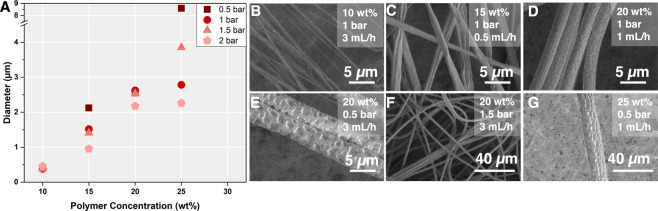
(**A**) The influences of pressure and polymer concentration are shown, for comparison, at a fixed flow rate of 1000 µL/h. A full data set is presented in Figs S4 and S5 with the histograms in Fig. S12. (**B**–**G**) SEM images demonstrating the tunability and uniformity of fiber diameters. The fibers show different shapes and surface morphologies which can be tuned with the polymer concentration and the air pressure.

A representative sample of different fibers obtained during this study is shown in Fig. 2. The upper and lower limits of the parameter ranges were determined to allow for continuous and smooth fiber fabrication and operation of the devices without clogging or instabilities. At very low polymer concentrations combined with low gas pressures, stable jetting could not be achieved and a dripping mode (followed by clogging from the drying polymer) was observed. For polymer concentrations of more than 25 wt%, the polymer solution was deemed too viscous, as it caused a pressure build-up inside the device which lead to rapid delamination of the nozzles. Furthermore, the handling of these solutions was difficult due to the fast transition from liquid to solid through slightest solvent evaporation (see Fig. S6). A detailed rheological analysis of the spinning solutions can be found in the supplementary information (Figs S6 and S7)^37^. Very high air pressures (>2 bar), especially for low polymer concentrations lead to jet breakup and irregularities in the fiber diameter. The fibers did not show conglutination at a collecting distance of 7 cm, confirming that the drying process is extremely fast and the fiber is fully formed within this distance.

Due to the varying mass-flows, the diameters of fibers produced in our device were strongly influenced by the polymer concentration (Figs 2(A) and S5). Low polymer concentrations (10 wt%) led to nm-diameter fibers (Figs 2(B) and S4(A)) and high polymer concentrations (25 wt%) to µm-diameter-fibers (Figs 2(G), S4(D), S5). Two additive effects contribute to this observation. Firstly, the lower initial polymer concentration will lead to a higher volume of acetone evaporation and, therefore, a thinner final fiber. Secondly, the lower concentration polymer solution has a lower viscosity compared to the high concentration polymer solution and is therefore more easily deformed by the gas flow-focusing. In other words, the fiber diameter depends on the liquid’s resistance to stretching (strain resistance) as well as its extensional viscosity and possibly also the elasticity which is present in many polymer solutions. The influence of the polymer concentration and shear on the f luid viscosity was confirmed by rheological data (Fig. S6).

The applied air pressure also affects the final fiber diameters obtained. A comparison of fibers obtained from the same polymer solution concentration and liquid flow rate, but at different gas pressures, shows that the diameters decrease with increasing gas pressure. Figures 2(A) and S6(C) shows fibers obtained from 20 wt% THV solution at varying air pressure concentrations. At a constant liquid flow rate of 1 mL/h, the fiber size decreases from 2.55 µm to 2.18 µm (applied pressure 0.5 bar and 2 bar respectively) with the air pressure increase. This effect is even more dramatic at higher flow rates, with the size of the fibers varying between 2.83 µm and 1.21 µm. The smaller jet has less mass and can therefore be accelerated by the gas to faster velocities which results in a stronger extensional flow and thinner fibers emerging from the nozzle. This behavior has also been described for liquid jets generated in GDVNs^39^ and is in line with the behavior described by Hofmann *et al*.^7^.

In summary, fiber diameters increase with increasing polymer concentrations and with decreasing gas flow rates. An overview heat map highlighting of these trends can be found in Fig. S9. Especially for the larger diameters (20–25% polymer solutions), the fiber diameters showed low relative standard deviations (Figs S10 and S11), showing that the controlled fabrication allows for high monodispersity of the sample. For lower polymer concentrations, these deviations were inflated, which is a repercussion of both the change in morphology (from round to flat fibers) as well as increased errors in the fiber diameter measurements arising from limitations in image resolution.

### Fiber shape and characteristics

**Figure 3:**
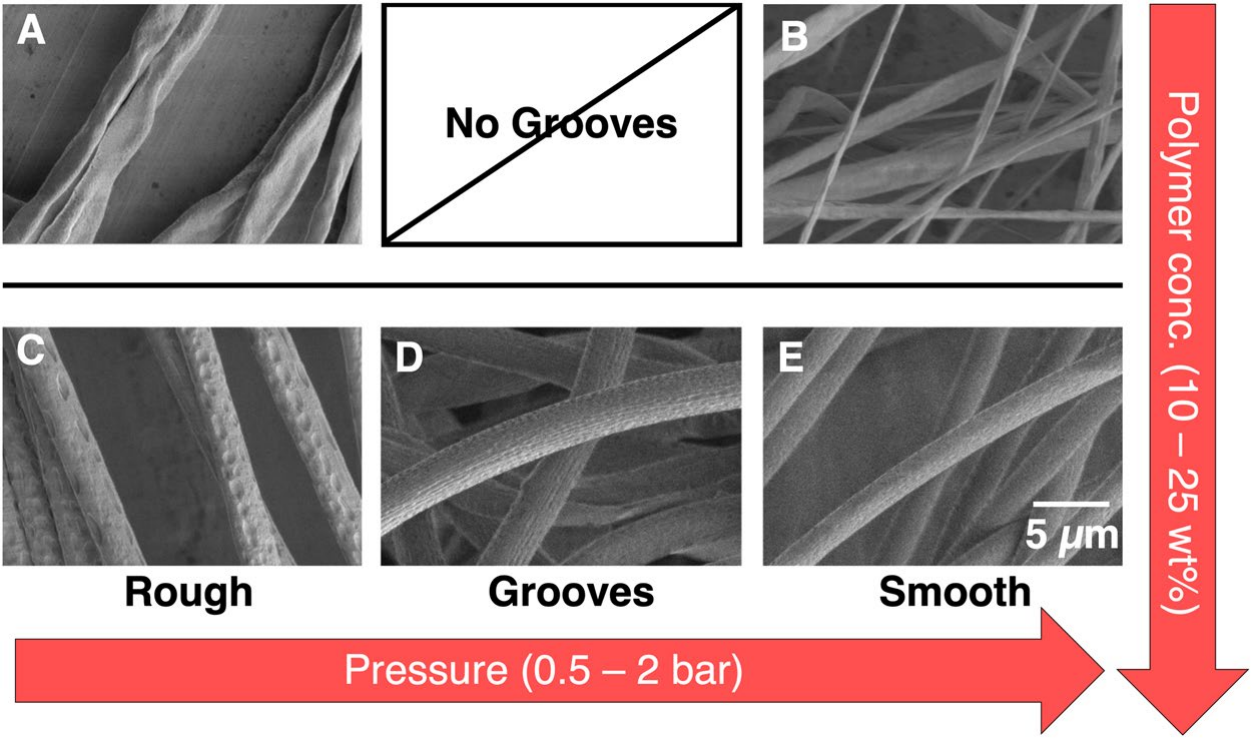
Table of the different fiber classes with representative SEM images. The fibers were categorized into flat (**A**,**B**, 10 wt–15 wt%) or round (**C**–**E**, 20 wt–25 wt%). Flat fibers were either rough or smooth (**A**,**B** respectively). Round fibers were rough, grooved, or smooth (**C**,**D**,**E** respectively). The shape was influenced by the polymer concentration and the surface roughness was controlled by the air pressure.

The polymer concentration and the air pressure do not only affect the diameter of the fibers but also the fibers’ overall shape and surface features. The fabricated fibers could be divided into five distinct classes according to their shape and surface morphology as shown in Fig. 3: flat-rough (A), flat-smooth (B), round-rough (C), round-grooved (D) and round-smooth (E). Figure 3 also shows how the classification is correlated with polymer concentration and gas focusing pressure used during fiber spinning. No beaded fibers, i.e. fibers with oscillating diameters along their length, were observed during our experiments, indicating a smooth and constant fiber formation process with rapid drying of the emergent fiber.

The overall shape of the fiber could be flat or round and was controlled by the polymer concentration. Lower polymer concentrations of 10–15 wt% produced flat fibers (Figs 3(A,B) and S4(A,B)), whereas round fibers were obtained from 20 wt% and 25 wt% polymer solutions (Figs 3(C,D) and S4(C,D)). Flattening of the fibers was caused by the device’s rectangular geometry and the asymmetric pressure profile at the gas-focusing region. The liquid flow channel is rectangular (15 µm × 40 µm w × h) and so is the nozzle opening (55 × 120 µm w × h, Fig. S3(F)). The gas channel envelops the liquid in three-dimensions but due to the nozzle dimensions, the liquid will experience higher compression forces in the horizontal direction which, in the case of the low concentration/low viscosity polymer solution, will dictate the asymmetry of the final fiber. An increase of this aspect ratio would potentially allow for the fabrication of flat, ribbon-like fibers with a high surface-to-volume ratios. This anisotropy effect is much less pronounced at higher polymer concentrations due to the higher viscosity and surface energy of the liquid resulting in circular fibers. Based on these observations we assume that a round (or square) nozzle geometry would be beneficial for the generation of round fibers.

**Figure 4:**
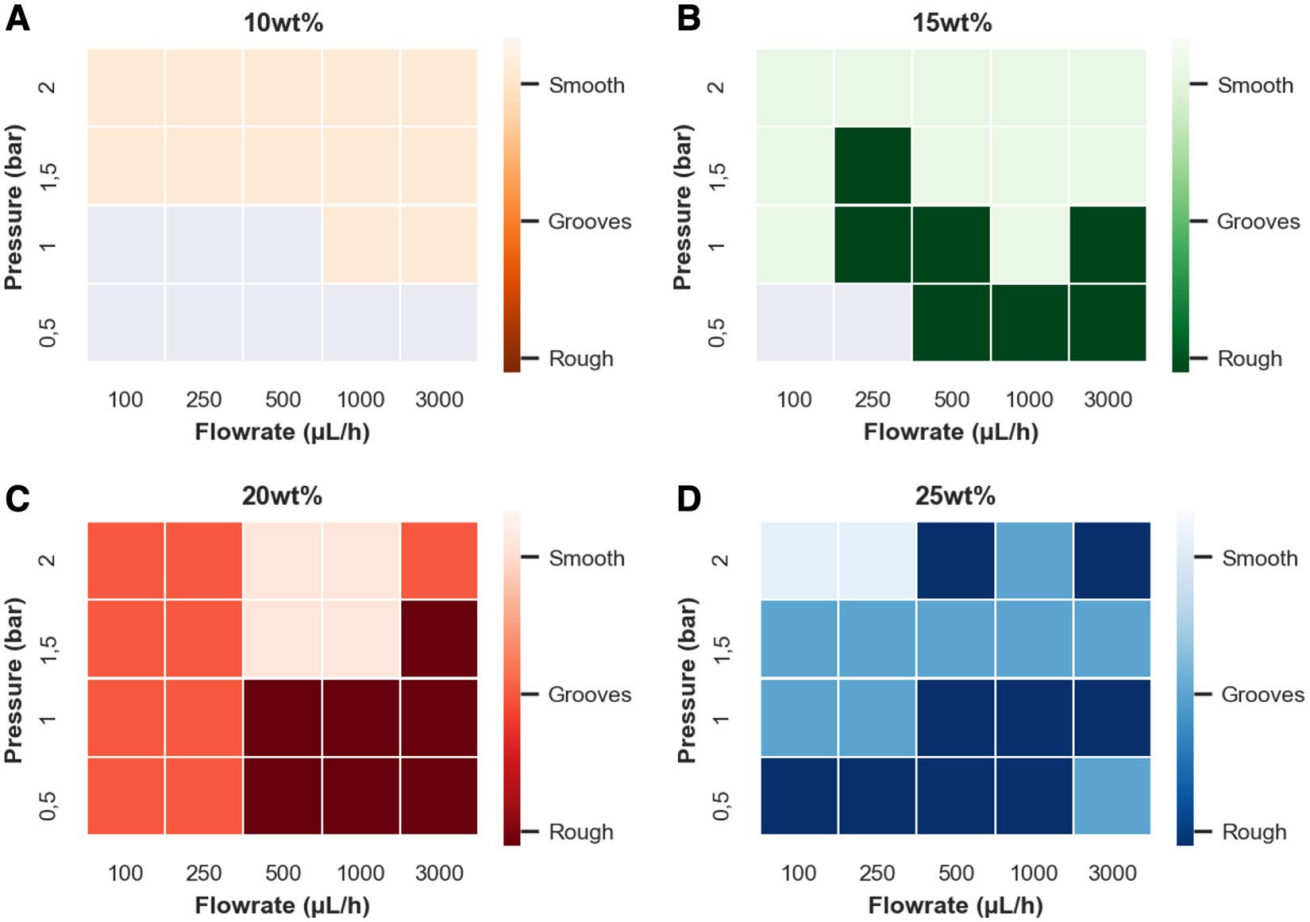
Fiber surface characteristics are influenced by the polymer solution concentration, flow rate and focusing gas pressure. (**A**) 10 wt% yields flat, smooth fibers. (**B**) 15 wt% polymer solutions lead to smooth and rough flat fibers with decreasing pressure. (**C**) 20 wt% and (**D**) 25 wt% lead to smooth, grooved or rough fibers, with decreasing air pressure. The detailed surface characteristics - smooth, grooved, and rough - are shown by the different color brightnesses. The corresponding SEM images and diameters can be found in the supplementary information (Fig. S4A–D). Grey areas in (**A**,**B**) display the “no jetting state”.

The produced fibers can be further classified by their surface morphology, as shown in Fig. 3. The most pro- nounced surface characteristics (e.g. craters and grooves) were found at high polymer concentrations, especially at 25 wt% polymer solution. Figures 4 and S4 show the morphology obtained from the selected combinations of flow rate, focusing gas pressure and polymer solution concentration.

At the lowest concentration of 10 wt%, mainly smooth and flat fibers were observed (Figs 4(A) and S4(A)). By increasing the polymer concentration to 15 wt% the flat fibers could be distinguished into flat-rough and flat-smooth fibers (Figs 4(B) and S4(B)). The flat-rough fibers formed at low pressures (0.5–1 bar) and showed small craters in the nm-range. At higher pressures (1.5–2 bar), the fibers showed only smooth surfaces. At high polymer concentrations of 20 wt% and 25 wt% fibers were classified into round-rough, round-grooved, and round-smooth, representing different levels of surface craters. The data suggests that the appearance of the different surface morphologies depends on the time scales and interplay between the solvent evaporation process at the surface and the velocity mismatch mechanism between the liquid jet and the air stream. The shear exerted on the surface of the liquid is coupled to the difference in speeds between the gas and the liquid surface. This shear is strongest in the converging flow-focusing region where the fast air flow accelerates the liquid. Our experiments show that round craters on the fibers’ surface have a higher tendency to be observed at lower pressures (e.g. 0.5 bar) as shown in Figs 3(C), 4(C,D) and S4(C,D). The appearance of similar craters has also been previously observed in fibers fabricated by electrospinning^40^. In electrospinning, surface porosity is achieved by a fast evaporating solvent in absence of an air stream and inducing phase separation during solvent evaporation, which divides the matrix into polymer-rich and polymer-poor regions as discussed further below^41^. At higher air pressures the fiber is stretched and grooves develop which are especially well expressed for the fibers spun with the 25 wt% polymer solution (Fig. S4(D)). At the highest pressure, the surface patterns can fully vanish resulting in a smooth surface (Fig. 4). Therefore, we believe that surface properties are mostly determined by the air pressure, or more precisely by the velocity of the air stream surpassing the liquid jet combined with a fast evaporating solvent. Interestingly, higher liquid flow rates led to an increase of surface roughness, but the mechanism for this is still unclear and needs further investigation.

The distinction between the different surface morphologies was done by evaluating SEM images of the fibers and is only a guide to the expected surface morphology vs. different experimental conditions. Of course, the change in morphology is continuous over the different experimental conditions and may vary especially in transition regions during the device operation. Further deviations from the expected morphology might be caused by changes in the environment (temperature or humidity).

### Inner morphology and porosity of fiber

**Figure 5:**
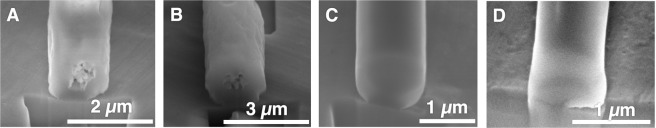
SEM images of THV fibers dissected with FIB showing the cross section of fibers produced at 2 bar, 3000 µL/h. (**A**) 10 wt% THV, porous, 1.2 µm diameter, (**B**) 15 wt% THV, porous, 1.6 µm diameter, (**C**) 20 wt% THV, solid, 1.05 µm diameter, and (**D**) 25 wt% THV, solid, 0.9 µm diameter.

The production of porous or micro-/nanofibers is of high interest for reducing their weight and increasing their surface to volume ratio. For example, such porous or grooved fibers can be obtained by electrospinning and controlling the polymer/solvent/anti-solvent interaction^42,43^. The underlying mechanism of such processes relies on the formation of pores upon the evaporation from the polymer-poor phase and solidification of the polymer-rich phase, which is also reflected by the phase diagrams of such ternary phases^42,43^. To investigate the inner structure of the here-produced fibers, focused ion beam (FIB)^44^ cuts were performed and revealed that the polymer concentration has indeed an impact on the fibers’ internal porosity. Fibers manufactured from polymer concentrations of 10 to 25 wt% at the same flow rate and pressure (3 mL/h, 2 bar) were investigated as shown in Fig. 5. Fibers spun with 10 and 15 wt% polymer solution showed core porosity (Fig. 5(A,B)) while the fibers spun from the 20 and 25 wt% polymer solution were completely solid (Fig. 5(C,D)). This observation is in agreement with recent literature^42,43^. At polymer concentrations ≤15 wt%, a larger volume fraction of solvent has to evaporate first before the fiber can solidify which in turn increases the drying time. It can also be assumed that the outside layer at the air interface dries first, while this initial layer could even slow the solvent evaporation from the fiber core even further. This combination gives the material enough time and mobility to separate into polymer rich and polymer poor phases, which represents a thermodynamically favored state. Fibers with and without inner pores have different physical properties. Inner porosity is desirable for fibers manufactured for biomedical applications (cell-laden fibers)^45^ or for fibers with increased thermal insulation properties while solid fibers can serve as materials for non-woven tissues or high-performance clothing. The possibility of tuning these properties as easily as changing the polymer concentration by 5 wt% allows access to these different applications as well as the possibility of making layered materials.

### Fiber spinning mechanics

**Figure 6:**
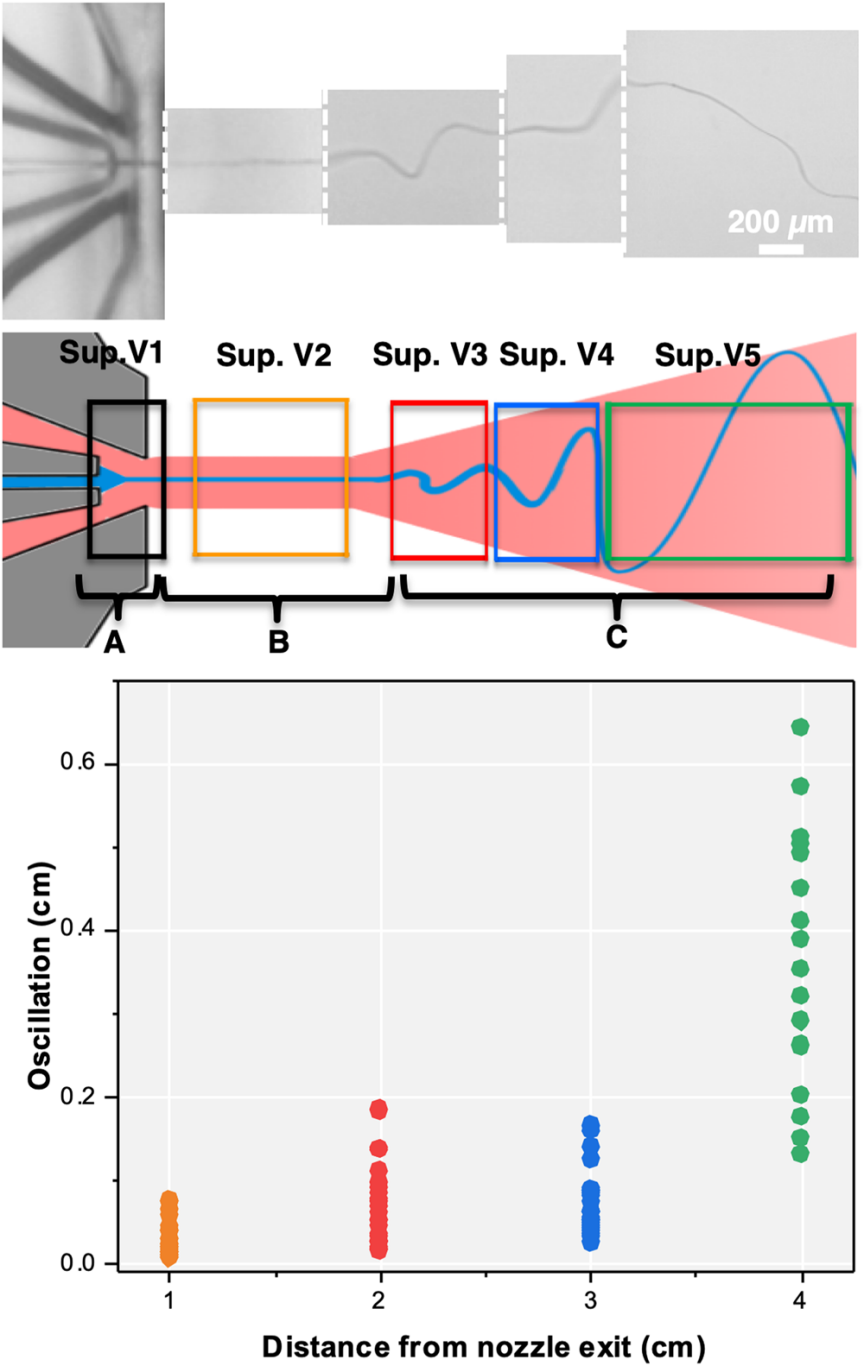
Schematic view of the fiber formation process using GDVN devices with (*Top*) high speed video microscopy snapshots. The white dotted lines indicate different highspeed video recordings. (*Middle*) An illustration of the hypothesis: The spinning process was separated into three sections, (**A**) gas focusing region, (**B**) jetting regime, and (**C**) thinning regime. (*Bottom*) The plot shows the oscillation amplitude of the emerging fiber during jetting perpendicular to the jetting axis.

Based on the experimental results obtained by SEM images and high speed video microscopy, a phenomenological hypothesis of the fiber spinning mechanics is suggested. This hypothesis divides the spinning process into three different regions as shown in Fig. 6: (1) the gas focusing region (2), the jetting regime and (3) the thinning regime. The gas focusing region was located inside the microfluidic device where the main and side channels intersect. The expelled liquid is flow-focused by the bypassing air stream and a solution cone geometry can be observed^46^. In this step, the jet diameter thins due to the hydrodynamic gas-focusing which is also the key step for the flow alignment of anisotropic particles. Subsequently, the liquid is ejected at the nozzle orifice as a straight jet entering the jetting regime, as seen from the very small oscillation of the emergent jet. This stable jetting behavior at 3 mL/h and 2 bar (20 wt% polymer solution) was roughly 1 cm. The length of this region depends on jet and air stream velocity as well as the viscosity of the solution. Within this region, the jet diameter is almost constant and only thinned by the loss of solvent due to evaporation. The end of the jetting regime is marked by the appearance of chaotic low-amplitude fluctuations which increase dramatically with the jetting distance and develop into a turbulent flow profile, causing the fiber to become thinner (thinning regime, 3). The turbulent behavior is characterized by the whipping of the fiber, as shown by the sudden increase of its oscillation amplitude. This behavior is caused by an increase of the Reynolds number (Re) and by Raleigh instabilities arising from the surface tension of the solvent. Even though the jet diameter is decreased within the hydrodynamic focusing region (1), the main fiber thinning process - especially for nanofibers at low polymer concentrations - seems to occur during the last stage due to the additional whipping or spiraling movement induced by the turbulent air flow. This behavior can stretch and thin the semi-solid fiber as it was observed by Benavides *et al*. where a comparable method was used^47^.

### Nanocomposite fibers

To demonstrate the versatility of this micro-/nanofiber manufacturing process, fibers impregnated with anisotropic hematite nanoparticles were also fabricated. 15% THV solutions with dispersed 650 × 65 (L/D) nm magnetic hematite nanoparticles (2.5 wt%) could be easily jetted under similar conditions as described before (1 mL/h and 1.5 bar). The rheological data of this spinning solution and the BSE-SEM image of resulting fibers can be found in Figs 1 and S7. The jetting behavior was smooth and continuous, suffering from no visible influences Due to the presence of nanoparticles. In fact, the spinning solutions showed a small shear thinning effect, possibly due to the shear alignment in a converging flow (Figs S6 and S7)^37^. Contrary to flat-smooth fibers expected under these conditions, the nanocomposite fibers were round and showed craters on the surface, indicating an influence on the surface morphology and overall fiber shape from the presence of nanoparticles. Rheology experiments showed, that the here used concentration of hematite nanoparticles did not significantly alter the rheological properties of the fluid (Fig. S7). BSE-SEM images of the nanocomposite fibers showed a uniform distribution of the particles inside the fiber (Fig. 1). Interestingly, these particles were also strongly aligned longitudinally along the jetting direction as shown in previous aqueous circular jets^48,49^. This strong alignment can be explained by the strong extensional flow field in the converging flow focusing region followed by a rapid solidification of the polymer. Due to this rapid fixation, any rotational diffusion of diverging flow is stopped which would otherwise influence the order parameter of these anisotropic particles^37,38^. Such nanocomposite fibers could find wide range of biomedical applications, for example in diagnostics (contrast agent), hyperthermia agent, drug delivery, or tissue engineering^10^.

## Conclusions

Here, we have reported a new microfluidic GDVN nozzle which allows continuous and reproducible fabrication of stable and uniform micro-/nanofibers with controlled surface morphologies and overall shapes. The influence of air pressure, polymer concentration, and flow rate on the fibers properties was investigated and revealed that surface roughness was controlled by a combination of air pressure and polymer concentration while the diameter and shape of the fibers were primarily influenced by the concentration of the polymer solution and marginally by the air pressure. A wide range of diameters ranging from a few hundred nanometers and up to ~15 µm were spun as endless single fibers using the same microfluidic device, which emphasizes the highly tunable operational nature of this technique. To our knowledge, this is the most gentle and simplest setup described to date for the fabrication of sub-micrometer fibers. Our extensive study of fiber morphologies shows a high tunability of their properties. The formation of rough, smooth, or grooved surfaces was attributed to the interplay of the evaporation process and the velocity (mis)match between polymer solution jet and its surrounding air stream. FIB-cut fiber cross sections also revealed that by simply changing the polymer concentration, fibers with different porosities could be obtained. These porous fibers have potential applications as scaffolds for tissue engineering, where cells could be loaded directly into fibers via gentler conditions compared to electrospinning.

Based on high speed video microscopy (Fig. 6), as well as SEM analysis of the fibers obtained, a hypothesis describing the fiber formation process was developed which connects the fiber diameter with both the gas flow-focused jet diameter and a further thinning regime. The facile fabrication as well as the operation of the devices provides a new and robust preparation procedure of microfibers. The devices can be re-used or, if contaminated, easily discarded due to their low fabrication cost. The gas-focusing geometry allowed for stable fiber jetting, meaning that these nozzles could be used to also directly coat surfaces in fiber, without the need of an intermediate spooling step. The current types of fibers produced have applications in various areas such as air/water filtration units, drug delivery systems, and cell growth studies. Further developments will enable the fabrication of more complex devices which can provide jet-in-jet environments for the fabrication of more sophisticated fibers with core-sheath, side by side or Janus-like structures. This jet-in-jet approach will also allow for the incorporation of clogging-prone additives, such as carbon nanotubes (CNTs) or other large, high aspect ratio nanoparticles. With this technique, the fabrication of advanced multifunctional/stimuli-responsive micro-/nanofibers, -wires or complex nonwoven structures can be achieved and are currently under investigation.

## Materials and Methods

SU-8 2050 photoresist and mr-Dev600 developer were purchased from Microchem Co. A MJB4 mask aligner from Süss MicroTec AG was used. All UV lithography steps were carried out in a clean room. Soft lithographic fabrication was carried out in a laminar flow box. 3-inch silicon wafers were purchased from Si-Mat Silicon Materials, polydimethylsiloxane (PDMS) Sylgard 184 kit from Dow Corning Co and 0.38 mm inner/1.09 mm outer diameter PE tubing was purchased from Scientific Commodities. THV-221GZ was obtained from Dyneon GmbH. High speed video microscopy was performed with a Phantom v711 camera (Vision Research Europe). The homogenization of the polymer-nanocomposite was performed using an UP400St sonotrode (Hielscher Ultrasonics GmbH).

### Photolithographic master template fabrication

The master template fabrication process was adopted from the previously described protocol^35^. Detailed fabrication steps can be found in the supplementary information (Fig. S1). In short, an SU-8 multilayer structure with the desired layer dimensions and channel geometries was fabricated using the negative photoresist SU-8 2050 on a 3-inch silicon wafer. Photolithographic emulsion film masks were designed using AutoCAD and used as UV-lithography patterns for the SU-8. Each layer was built in turn to define the final three-dimensional structure of the device.

### Microfluidic device fabrication

The patterned wafer was used as the master template for producing PDMS-based molds. The PDMS and curing agent were mixed with a weight ratio of 10:1, poured onto the patterned SU-8 wafer and degassed to remove air bubbles. The polymer was cured at 75 °C for 2 h, peeled off the SU-8 master and inlet ports were punched into the polymer using a 0.75 mm biopsy puncher. The outlet nozzles were carefully cut using a razor blade under a microscope. The two device halves were washed with isopropanol and cleaned with compressed air, thoroughly air-dried and activated using O_2_ plasma (10 W, 0.38 mbar, 100 s). Complementary device halves were bound at 45 °C for several hours to yield three-dimensional microfluidic nozzles (see Fig. S1).

### Spinning procedure and analysis of fiber properties

The thermoplastic fluoropolymer THV 221 GZ (THV) was obtained as pellets and used without further purification. 10, 15, 20, and 25 wt% THV solutions in acetone were prepared by slowly stirring until complete dissolution at RT. The polymer solution was loaded into a 10 mL syringe and connected to the liquid channel and a compressed air inlet to the gas channels of the spinning device, both using 1.09 mm outer diameter (OD) PE tubing as shown in Fig. S2. To start the spinning process, air flow was initiated first and stabilized at 0.5–2 bar pressure difference followed by the polymer flow which was controlled using high-precision neMESYS syringe pumps at a flow rate of 100–3000 µL/h. The fibers were collected on substrates covered in aluminum foil, which were placed 7 cm away from the nozzle. Optical microscopy images were taken during the fiber fabrication process using and inverted optical microscope coupled to a high speed camera at magnifications of 4x, 10x and 20x. The fibers collected on aluminum-coated substrates were directly used for SEM analysis while avoiding any sample alterations. SEM images were usually taken at 1000x and 5000x magnification. The determination of the fiber diameter was done manually with ImageJ at random points of the SEM image to assure a statistical distribution. Further, the diameters of the flat fibers were measured at their thickest point. The mean fiber diameter and the standard deviation were calculated using the arithmetic mean.

### Nanocomposite fiber spinning

3 mL of a 30 wt% polymer solution of THV 221 GZ in acetone was stirred overnight. Shortly before preparing the spinning solution, the Fe_2_O_3_ hematite nanoparticles (650 × 65 nm, l × d)^50^ were dispersed in 1 mL of acetone in a tall and thin 2.5 mL vial. The dispersion was then homogenized with a sonotrode and mixed in a 1:1 v/v ratio with the 30 wt% THV 221 GZ polymer/acetone solution to obtain a final 15 wt% polymer solution with a 2.5 wt% concentration of nanoparticles. Due to the relatively high viscosity of the polymer solution, sedimentation could not be observed during weeks when the composite solution was stored in a shelf.

## Supplementary information

Supplementary Information
