# Supplementary material for: Solution blow spinning of polymer/nanocomposite micro-/nanofibers with tunable diameters and morphologies using a gas dynamic virtual nozzle
Source: Sci Rep. 2019 Oct 4;9:14297. doi: 10.1038/s41598-019-50477-6 (PMC6778068; doi:10.1038/s41598-019-50477-6)
Supplement: Supplementary file 1 — Supplementary Information [file 41598_2019_50477_MOESM1_ESM.docx]

**Supplementary Information for**

Solution blow spinning of polymer/nanocomposite micro-/nanofibers with tunable diameters and morphologies using a gas dynamic virtual nozzle

Ramakrishna Vasireddi**^†^**, Joscha Kruse**^†^**, Mohammad Vakili, Satishkumar Kulkarni, Thomas F. Keller, Diana C.F. Monteiro and Martin Trebbin*

^†^These authors contributed equally

* Corresponding author:

Martin Trebbin

Department of Chemistry, The State University of New York, University at Buffalo,
760 Natural Sciences Complex, Buffalo, New York 14260-3000, USA

Phone: +1 716 645 4274

Email: mtrebbin@buffal.edu

**This PDF file includes:**

Supplementary text

Caption for movies V1 to V5

Figs. S1 to S13

**Other supplementary materials for this manuscript include the following:**

Movies V1 to V5

**Supplementary Information Text**

**Device Fabrication.** (A) A layer of SU-8 was spin coated on a silicon wafer. The thickness was controlled through the rotational speed and duration of the spin coating program. (B) The wafer was pre-baked until the solvent was evaporated to solidify the photoresist. The SU-8 layer was structured by exposure to UV light through a photolithographic mask (C). The choice of a positive or a negative photoresist determines whether the areas exposed to UV-light will degrade or crosslink. SU-8 is a negative photoresist. After UV-irradiation, the wafer was post-baked to fully crosslink the exposed structures (D). Steps (A)-(D) were repeated as many times as layers are desired. As last step of the lithographic production process, the wafer was developed in mr-Dev600, which removed only the non-cross-linked photoresist (E). The wafer was then used as a stamp for the soft lithographic fabrication steps (G)-(I). First PDMS and curing agent are mixed in a 10:1 weight ratio, poured onto the master and degassed. The ratio between PDMS and the curing agent determines the stiffness of the material. This process crosslinks the PDMS, yielding a solid but flexible structure (G). The cured PDMS was then cut into the desired nozzle halves under a microscope. Special attention was given to the nozzle exit cuts. As last step, the bottom and top halves were bonded to each other after plasma activation (H).

**Movies V1-V5.** The movies are the highspeed camera video recordings mentioned in Fig. 5 where one can also find at which position each movie was recorded at. It is important to note that the depth of field was very shallow (e.g. sharp images in V1-V2) which clearly shows the fiber’s spiraling above and below the focusing plane (e.g. in V3-V5).


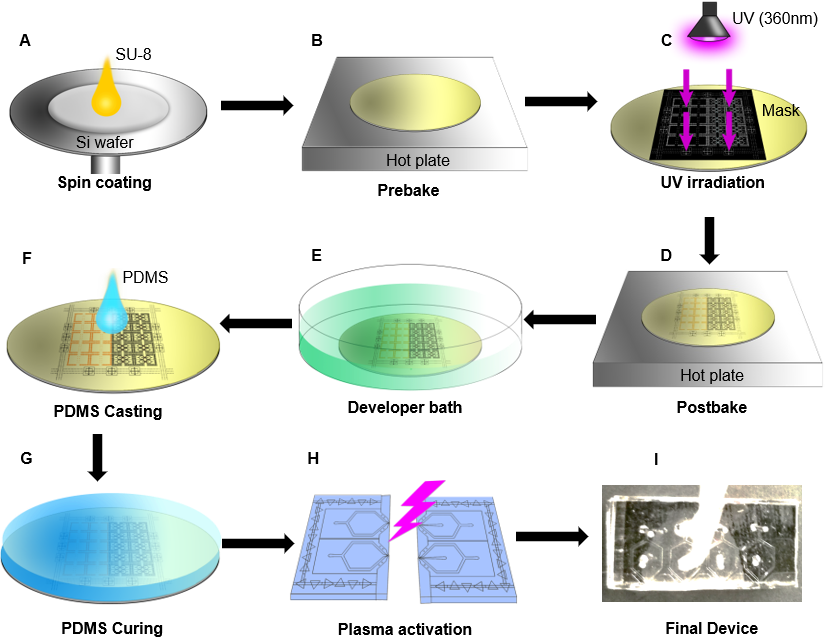


**Fig. S1.** Schematic representation of the microfluidic liquid fiber jet device fabrication steps using standard photolithography and soft lithography techniques.


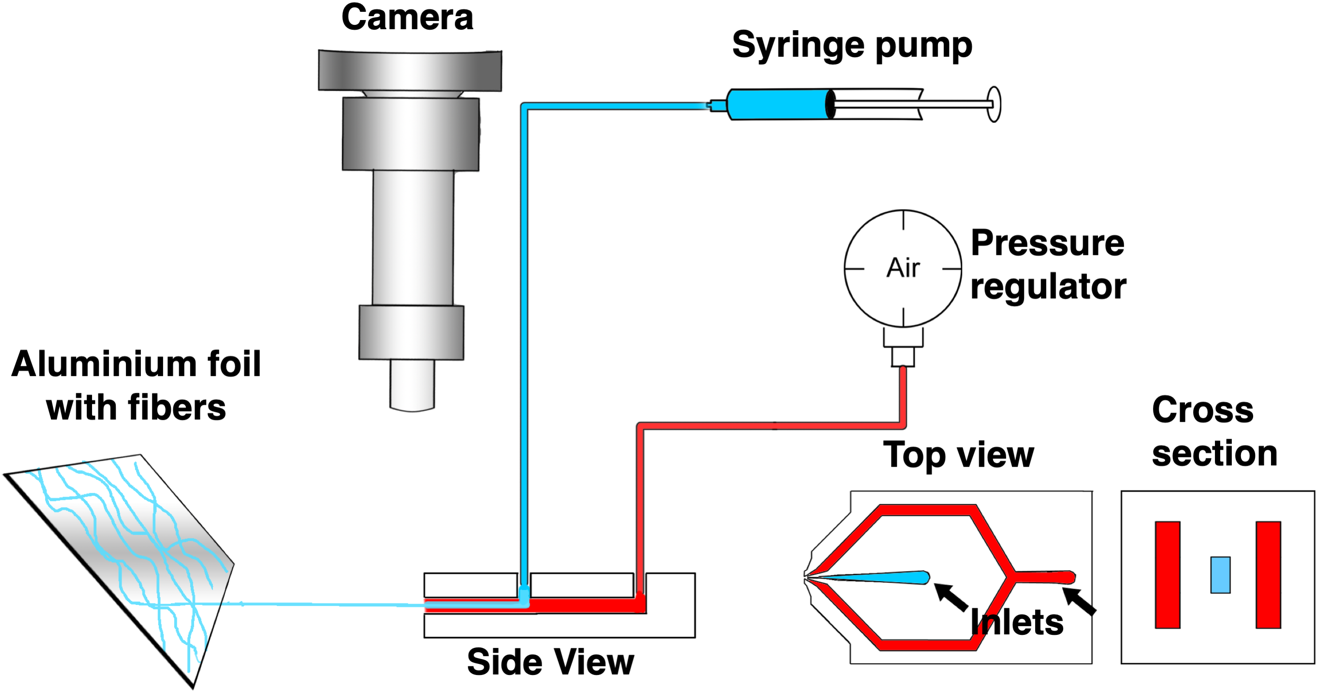


**Fig. S2.** Schematic view of the experimental setup for the analysis of the fibers. The microfluidic nozzle was attached to pressurized air and the polymer solution. While operating, the nozzle and the jet were observed by a high speed camera. The fibers were collected on a substrate covered in aluminium. The bottom right corner shows the top view and the cross section of the three-dimensional gas focusing region of the nozzle. Air channels are marked in red and solution channels are marked in blue.


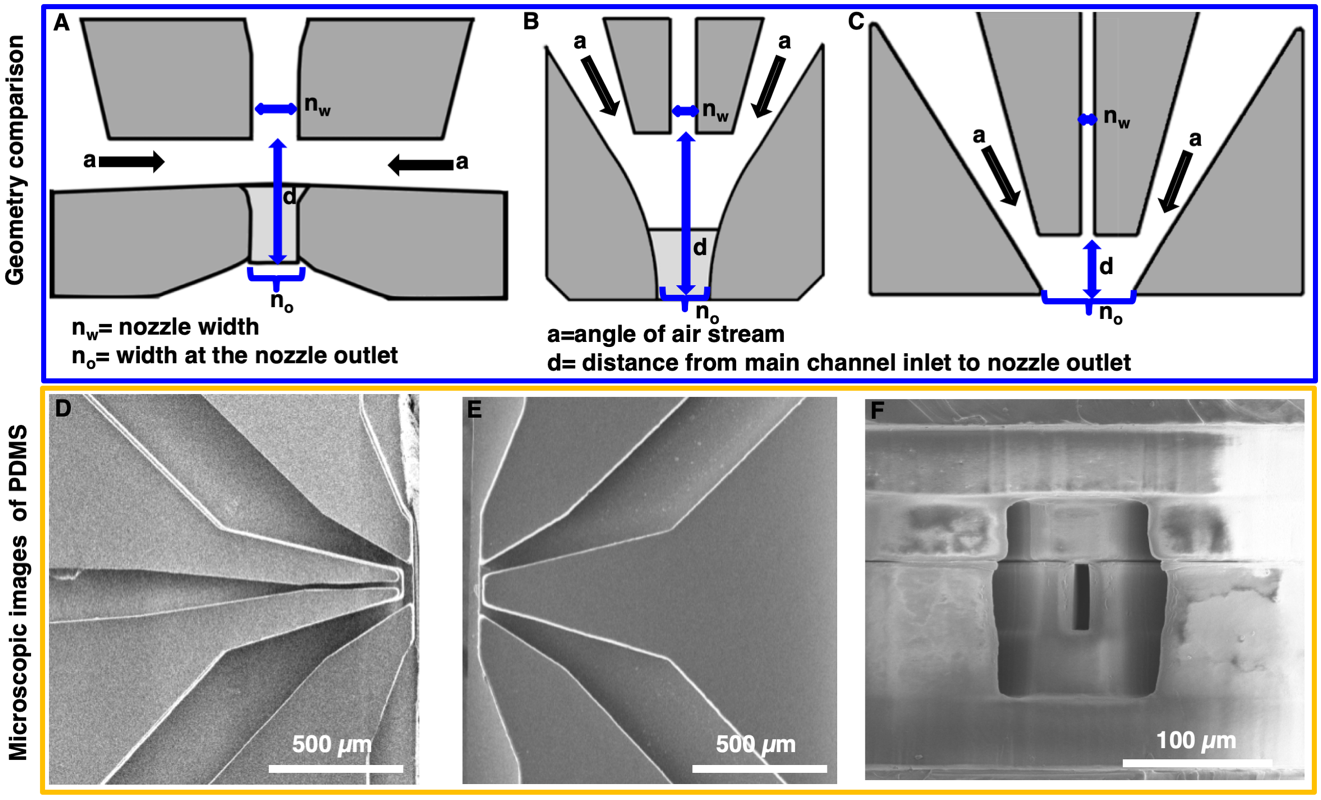


**Fig. S3.** (*Top*) Comparison of the geometries of previous microfluidic GDVN designs. (A) Microfiber spinning nozzle by Hofmann *et al*. (7); n_w_ = 30 µm, n_o_= 30 µm, a= 90^o^, d= 80 µm, (B) GDVN for liquid jets by Trebbin *et al*.(35); n_w_ = 15 µm, n_o_= 30 µm, a= 15^o^, d= 95 µm, (C) Nanofiber spinning nozzle developed for this work; n_w_ = 15 µm, n_o_= 55 µm, a= 15^o^, d= 35 µm, (*Bottom*) SEM images show the (D) upper and (E) lower PDMS halves (F) SEM image of the asymmetric nozzle outlet.


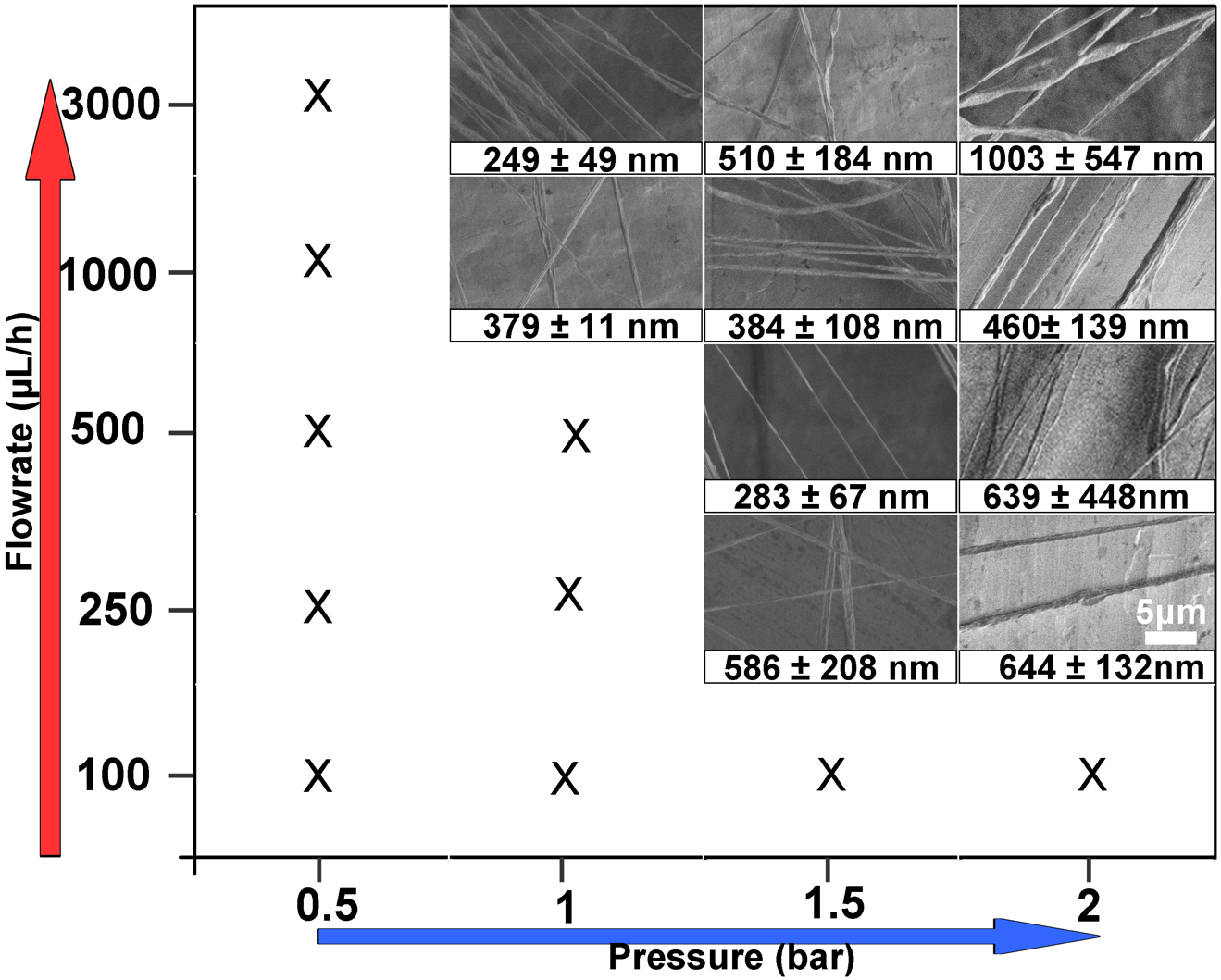


**Fig. S4 A).** SEM images of fibers w.r.t flowrate vs pressure at (A) 10% polymer concentration.


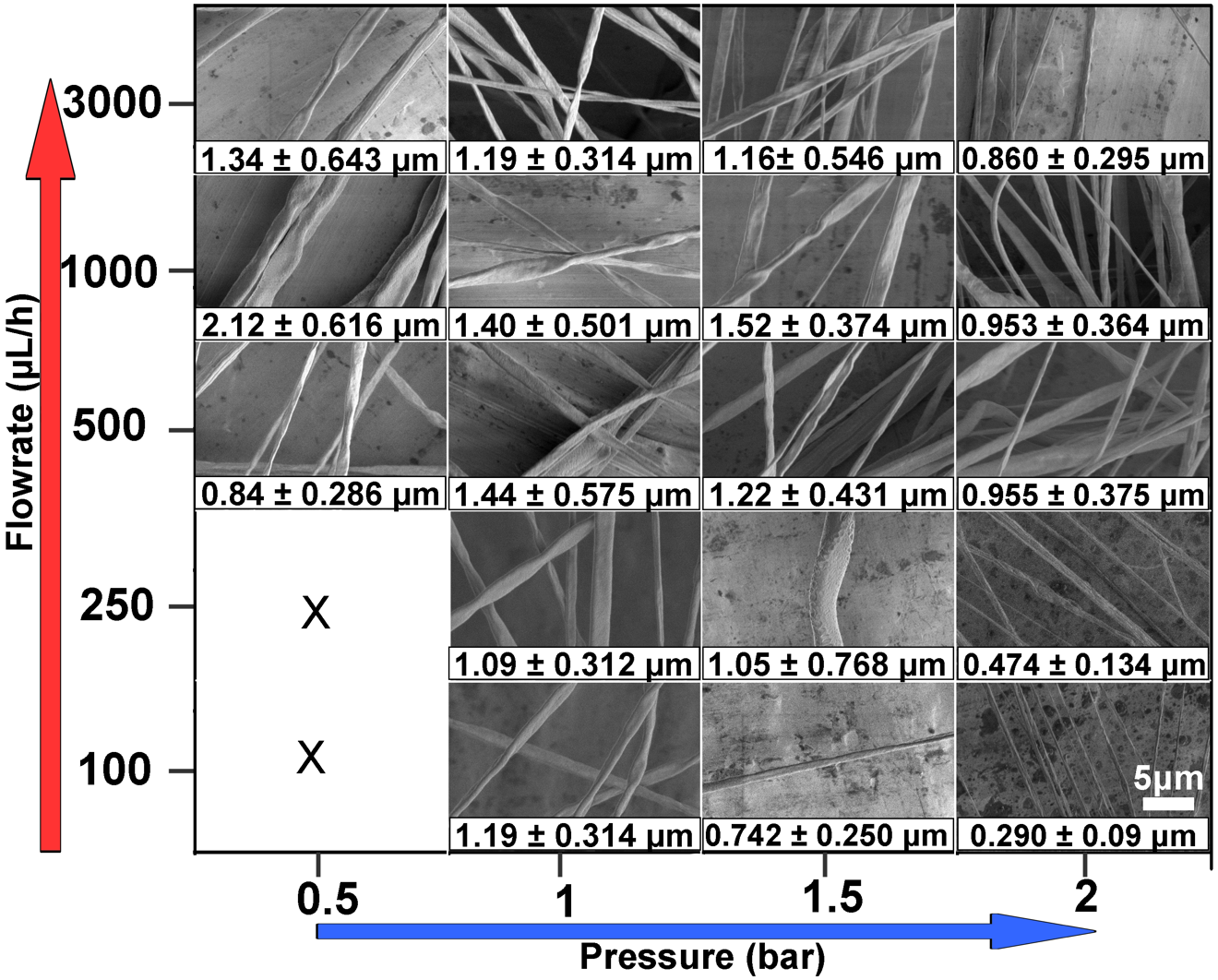


**Fig. S4 B).** SEM images of fibers w.r.t flowrate vs pressure at (B) 15% polymer concentration.


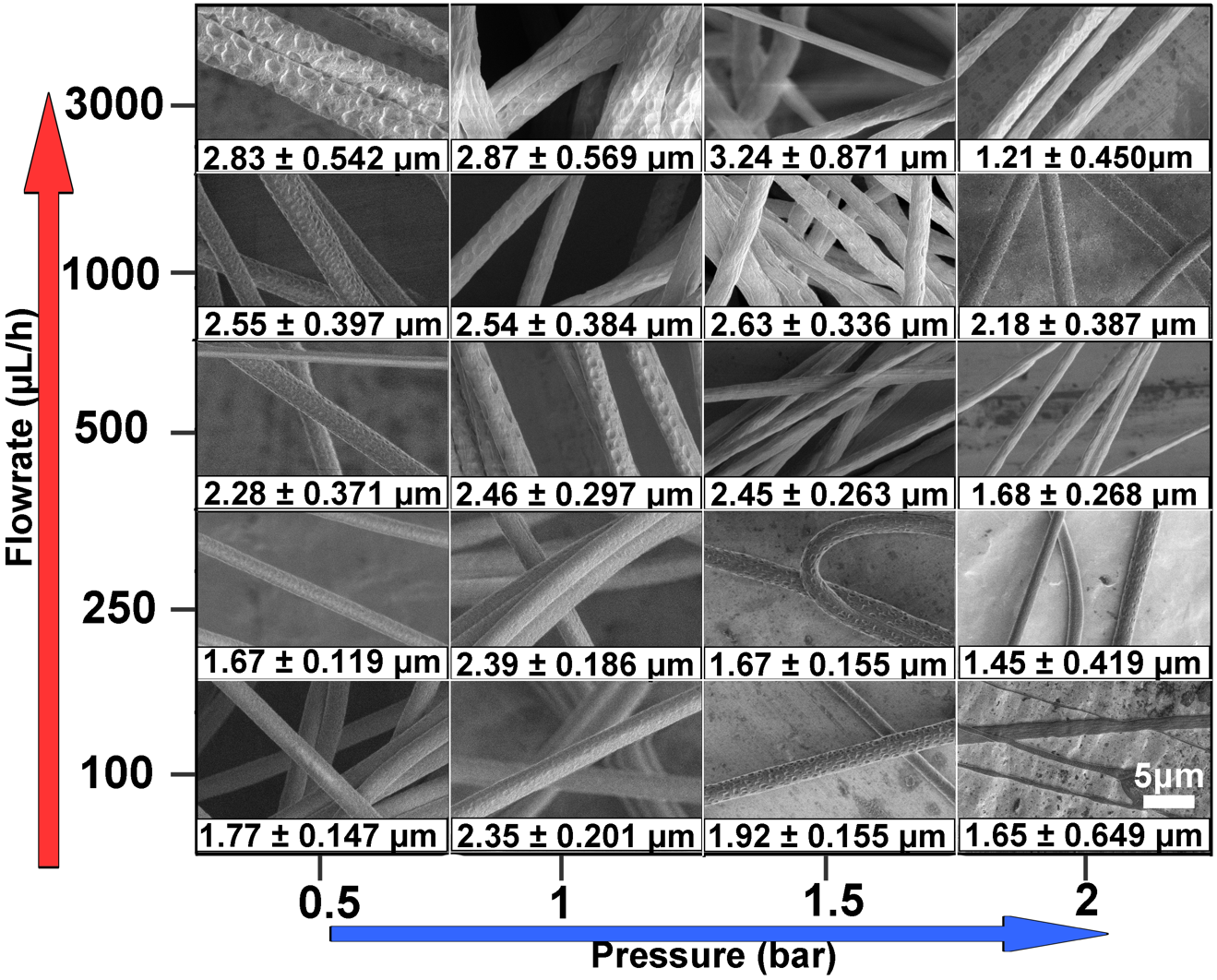


**Fig. S4 C).** SEM images of fibers w.r.t flowrate vs pressure at (C) 20% polymer concentration.


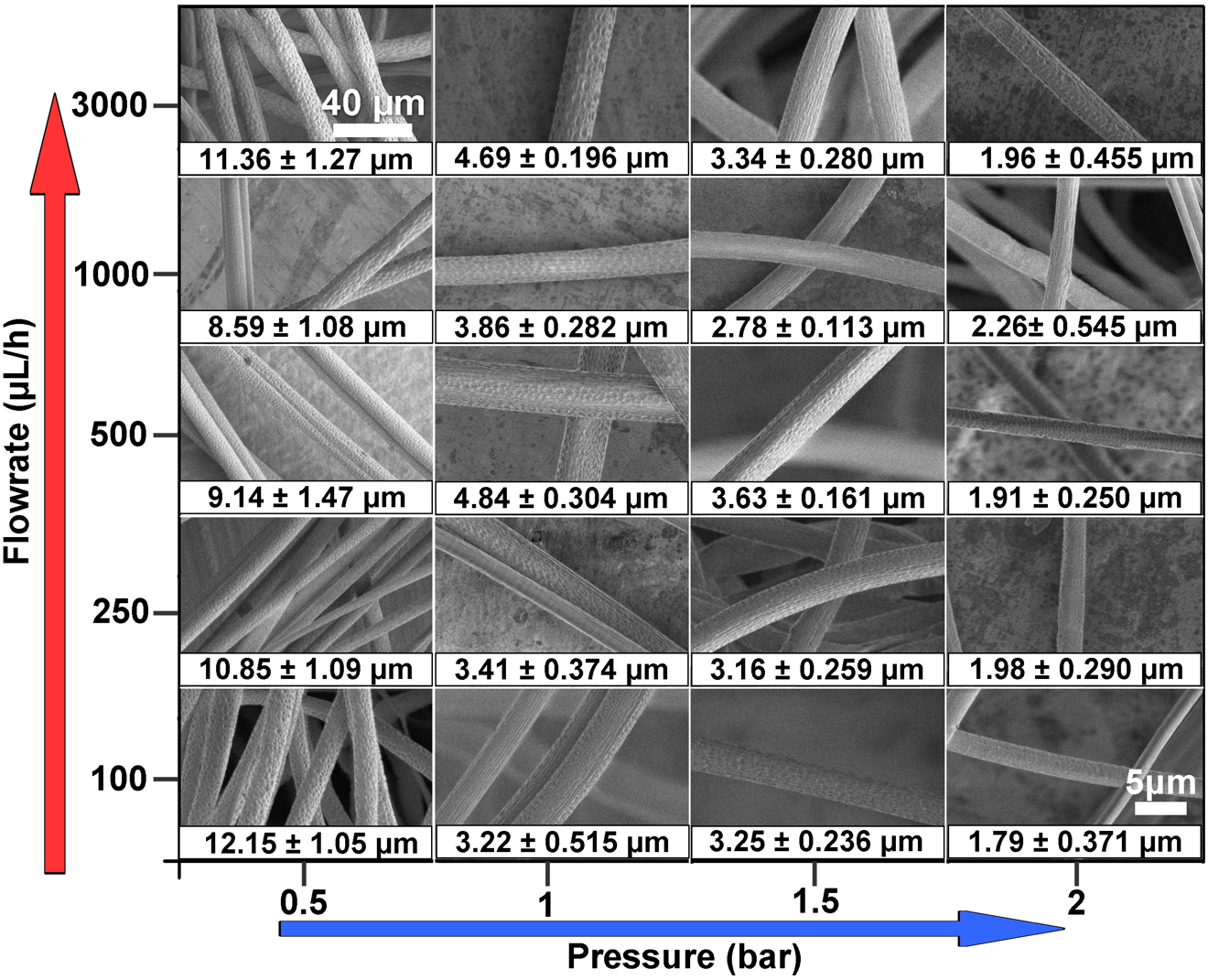


**Fig. S4 D).** SEM images of fibers w.r.t flowrate vs pressure at 25% polymer concentration. The 40 µm scalebar goes for 0.5 bar column, the 5 µm scalebar for the rest.

**
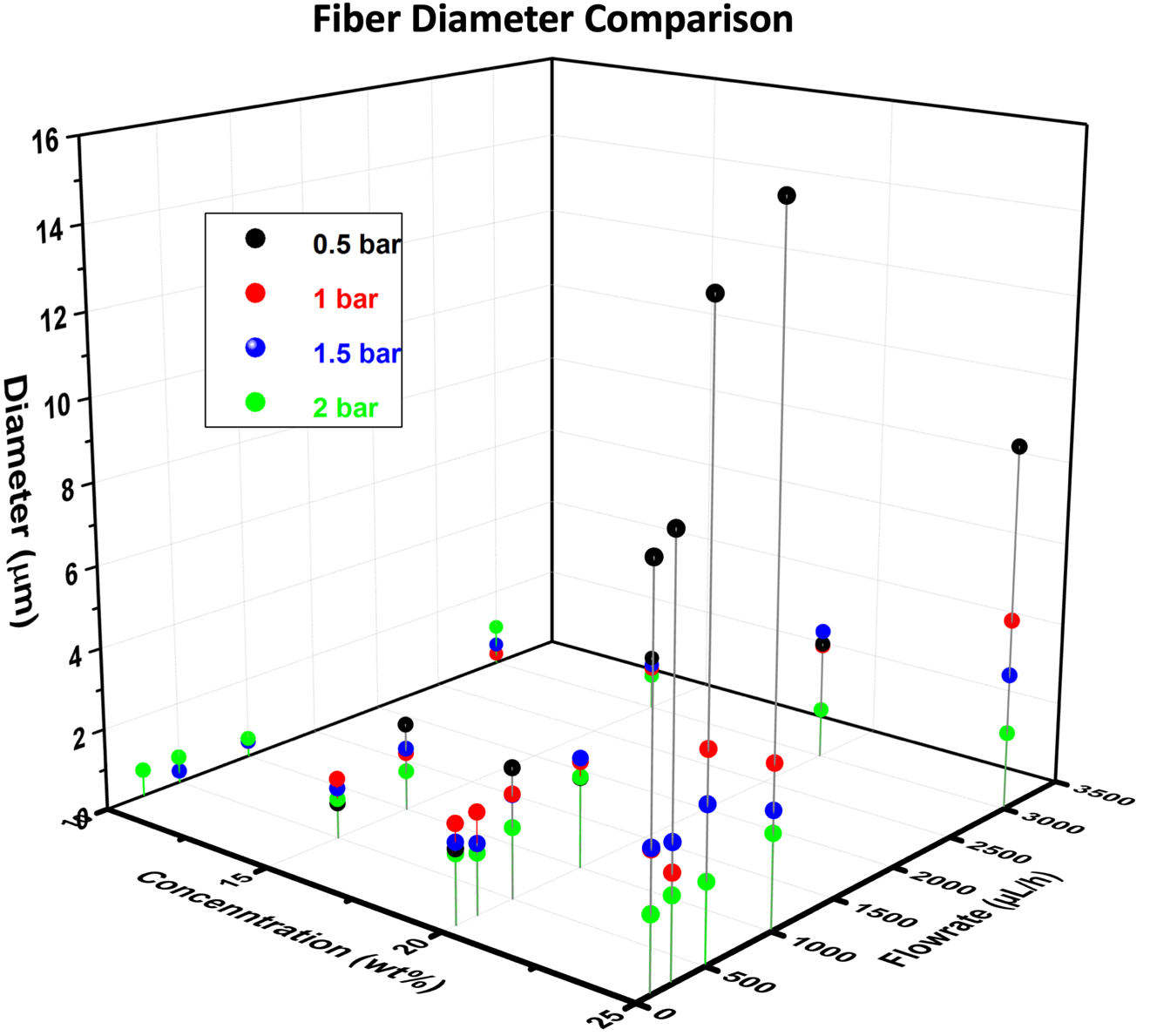
Fig. S5.** Diameter measurements of all parameter combinations investigated. Several trends are caused by changes of flow rate, concentration and air pressure are demonstrated here. First, the diameter increases with an increasing polymer concentration (from left to right), especially between the transition from 20 wt% to 25 wt%. Second, with increasing air pressure the fiber diameters decrease (see color coding). Furthermore, the influence of air pressure was greater at higher polymer concentrations. Lastly, the flowrate has only little influence in comparison to polymer concentration and air pressure (from front to back) although it can affect the stability of the flow-focused jet.

**
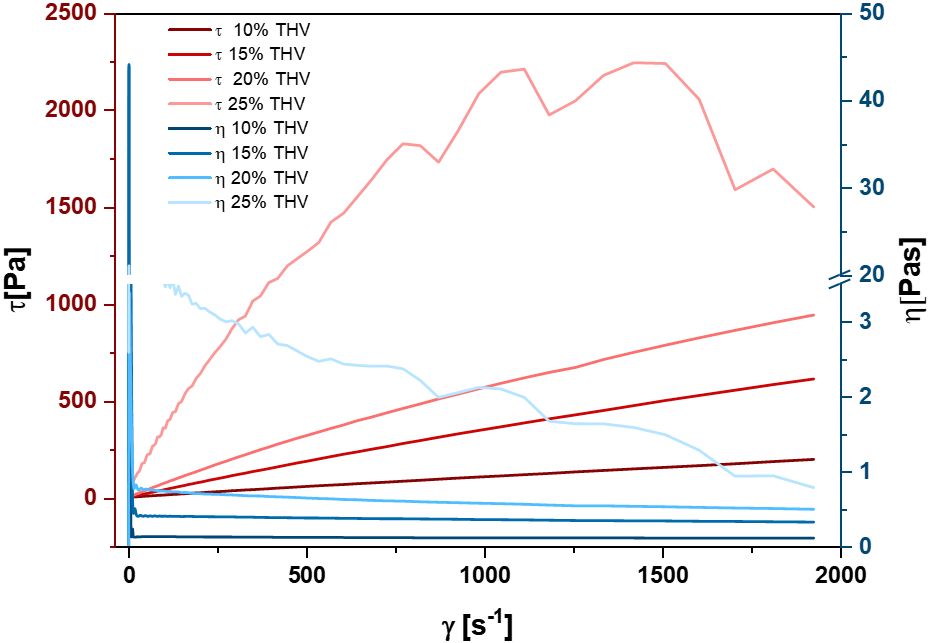
**

**Fig. S6.** Rheology data for THV 221 GZ polymer solutions in acetone from 10 wt% to 25 wt%. All concentrations of polymer solutions show a linear trend. The viscosity decreases slightly with an increasing shear rate (shear thinning), except for the highest polymer solution (25 wt%) where the graphs follow the same trend but not in a linear way which might be caused by fast solvent evaporation. Unfortunately, the molecular weight of this commercially-available polymer was not disclosed. Further information considering the material properties can be found with the link: https://multimedia.3m.com/mws/media/688833O/td-thv-221gz-eng.pdf (Accessed April 4^th^ 2019).

**
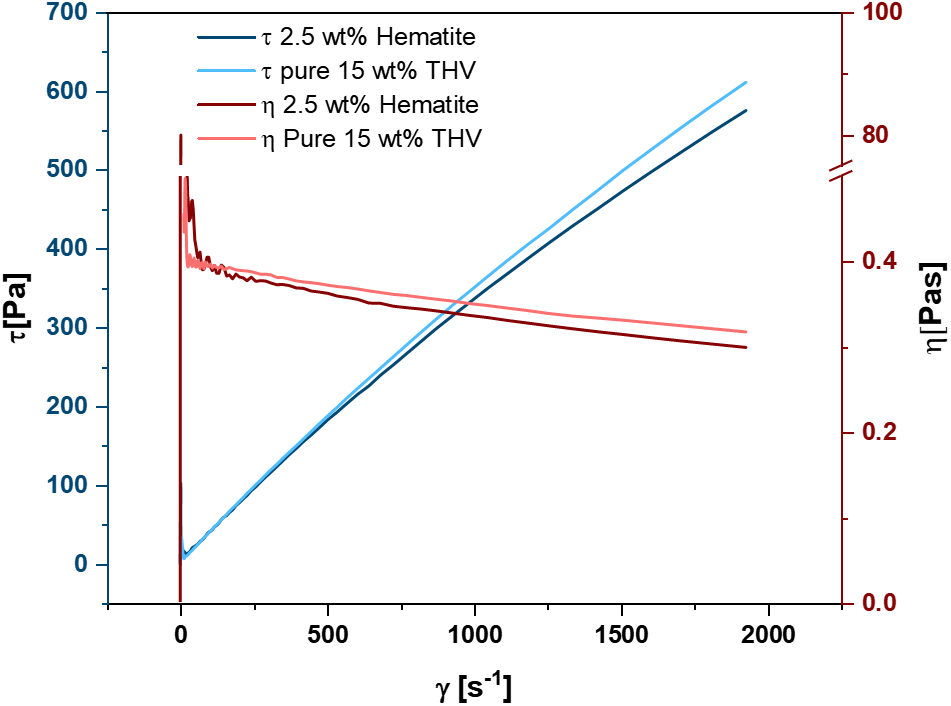
**

**Fig. S7.** Rheology data for 15 wt% THV 221 GZ polymer solutions in acetone with (dark colors) and without (bright colors) hematite nanoparticles. These anisotropic particles show only slight influence on the viscosity of the spinning solution. In fact, a small shear thinning effect could be observed - possibly due to the shear alignment in a converging flow (Trebbin *et al.*, *PNAS* **2013**, *110*(17), 6706-6711. DOI: 10.1073/pnas.1219340110). Due to the relatively high viscosity of the polymer solution, sedimentation could not be observed during weeks when the composite solution was stored in a shelf.

**Gas flow rate analysis.** To estimate the gas flow rate as a function of the applied pressure, we submerged the microfluidic device slightly under water and measured the time until 25 mL of gas are collected in a reservoir. The measured values are in a similar range as the values measured for comparable GDVN-devices described earlier. (Trebbin *et al.*, *Lab Chip* **2014**, *14*, 1733. DOI: 10.1039/c3lc51363g)





**Fig. S8.** Change of the gas flow rate as a function of the applied pressure.

**
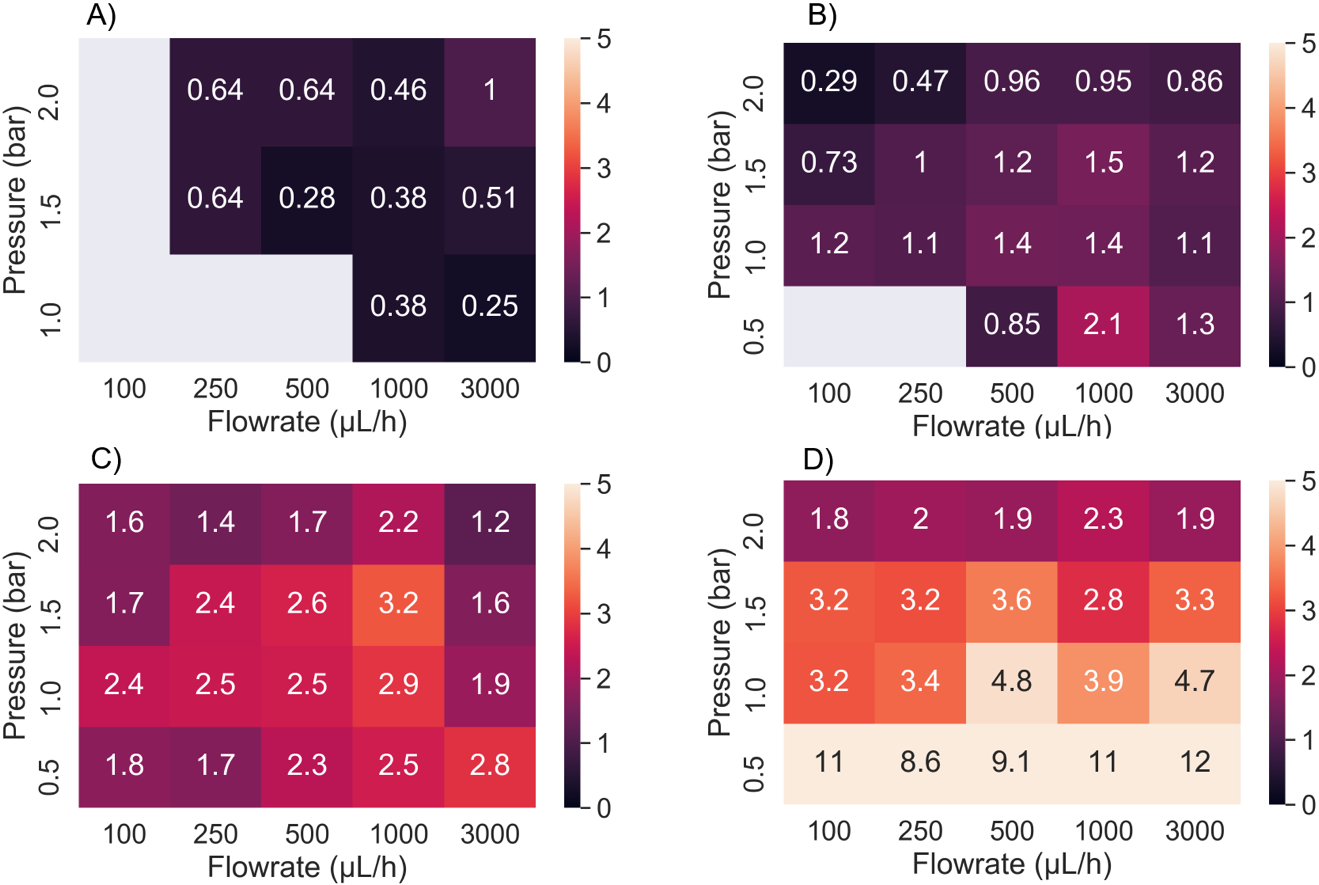
**

**Fig. S9.** Heatmap of the fiber diameters (values in µm) for 10 wt% - 25 wt% polymer solution A) - D). The map clearly shows that the diameter increases with increasing concentration and decreases with increasing pressure. The chosen colorscale robustly represents 90% of the data, with the exception of the largest fibers (25% polymer, 0.5 bar) which are outside of the scale.

**
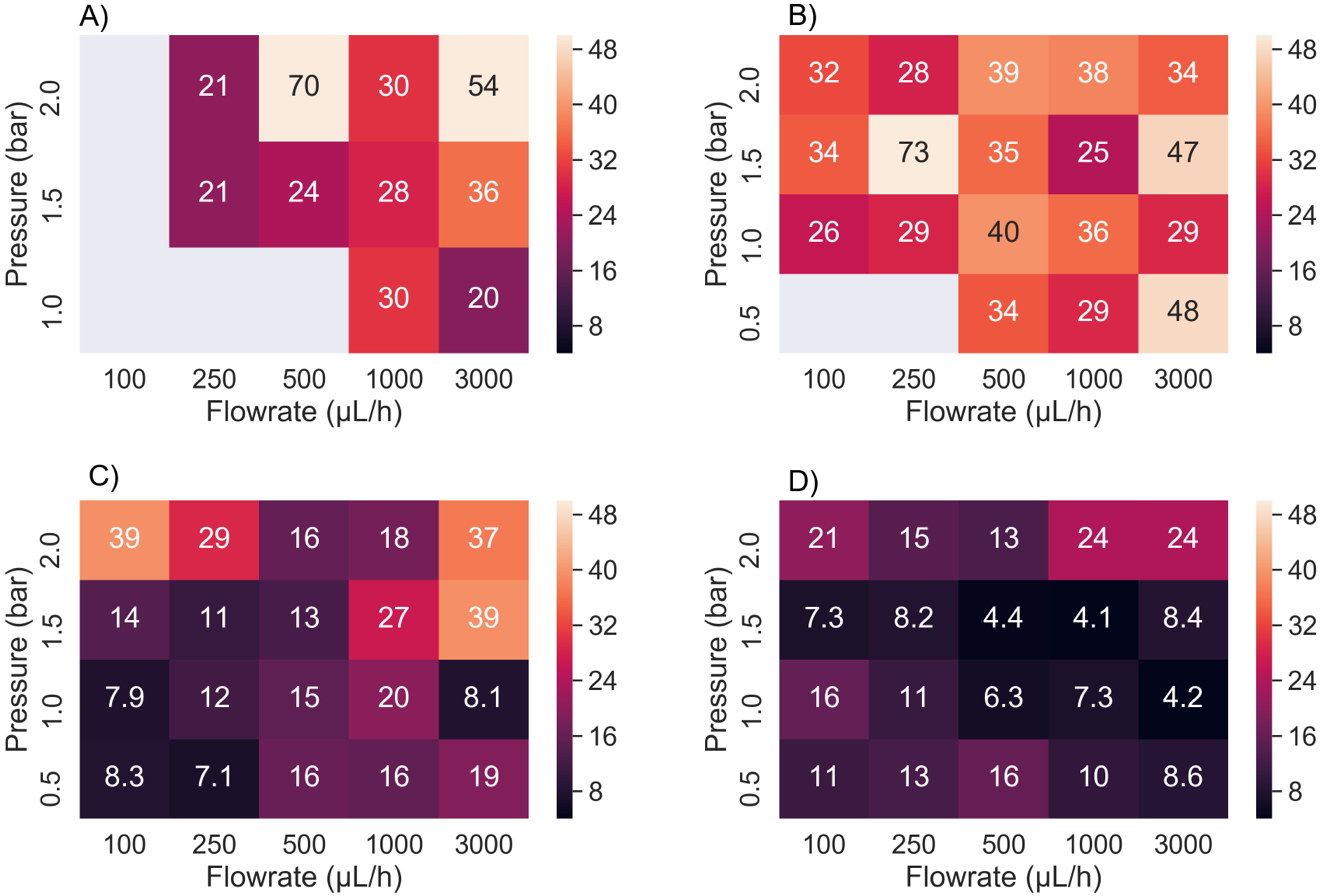
**

**Fig. S10.** Heatmap of the relative standard deviation of the fiber diameters (values in %) for concentrations of 10 wt% – 25 wt% polymer solutions A) - D). The relative standard deviation is much bigger for flat fibers, which arises from their nonuniformity. The chosen color scale robustly represents 95% of the data, with the exception of the 3 largest values which are outside of the scale.

**
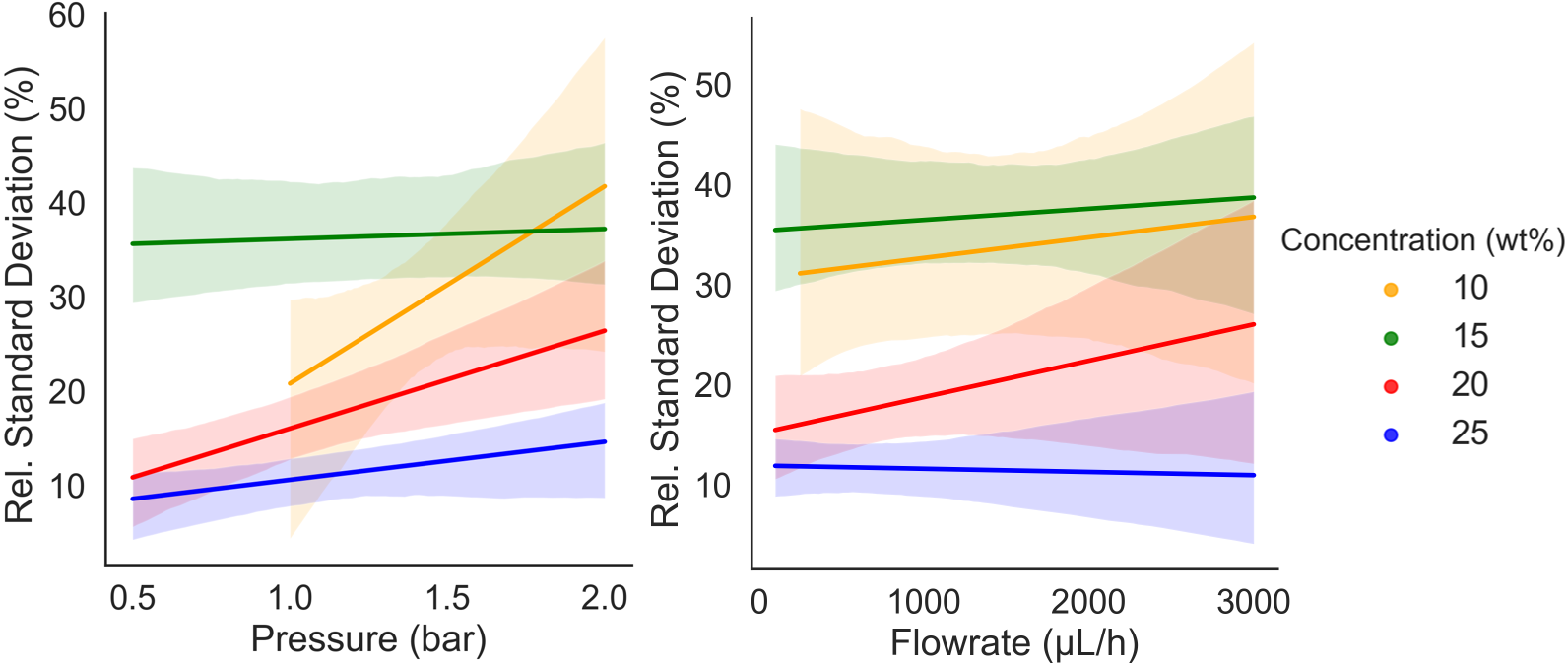
**

**Fig. S11.** Comparison of standard deviation for all measured fibers. First, the relative standard deviation (stdv%) drops with higher polymer concentrations, especially between 15 wt% and 20 wt%, where a large decrease can be found. This is probably due to the geometrical transition from flat to more uniform round fibers. Furthermore, by increasing the pressure, the stdv% is also increased, a trend that was can be also seen with respect to the flowrate, which was reportedly previously (**1).** J. E. Oliveira, E. A. Moraes, R. G. F. Costa, A. S. Afonso, L. H. C. Mattoso, W. J.Orts and E. S. Medeiros, J. Appl. Polym. Sci., 2011, 122, 3396–3405). Although, the variance increased at higher flowrates.


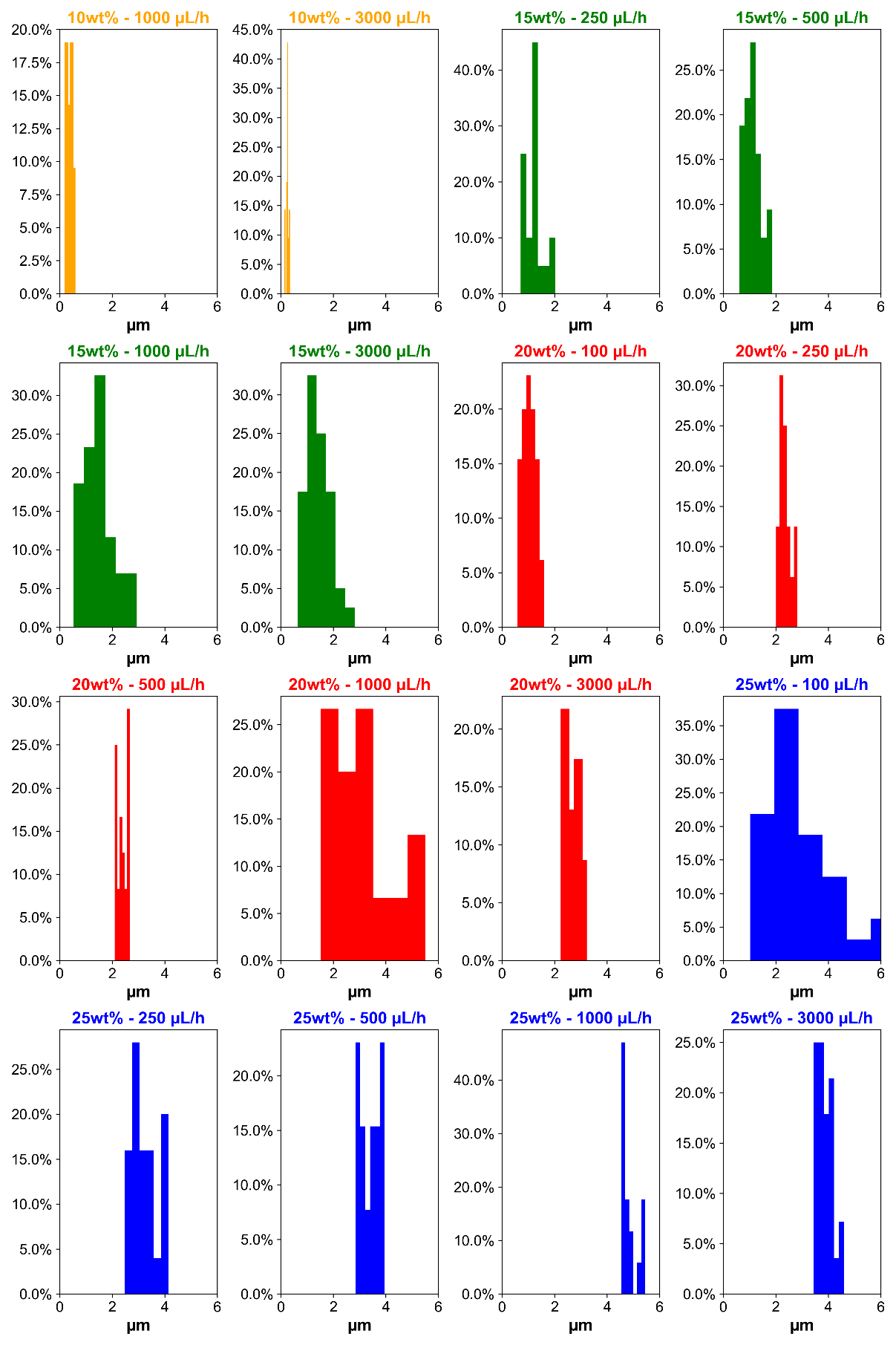


**Fig. S12.** Histograms for fiber diameters from Fig. 1A (1 bar). Y-axis shows the percental distribution and x-axis the diameter in µm.
